# A 15-Year-Old Female Presenting With Traumatic Diaphragmatic Hernia One Year After a Car Accident

**DOI:** 10.7759/cureus.24141

**Published:** 2022-04-14

**Authors:** Raymond C Winstead, Varun Kumar

**Affiliations:** 1 Pediatrics, East Tennessee State University Quillen College of Medicine, Johnson City, USA

**Keywords:** diaphragmatic hernia, pediatric, traumatic diaphragmatic hernia, delayed diaphragmatic hernia, case report

## Abstract

Traumatic diaphragmatic hernia (TDH) is a known complication in patients with abdominal injuries. Delayed TDH, which presents long after the traumatic event, is a rare subset and is often missed upon initial presentation. We discuss a case involving a 15-year-old female who presented with persistent nausea, vomiting, and epigastric pain and was subsequently diagnosed with TDH via chest x-ray, later confirmed by CT scan. Further investigation of the patient’s history revealed a motor vehicle accident one year prior in which the patient sustained an injury to the left chest. A chest x-ray at that time did not show signs of a diaphragmatic hernia. Surgical evaluation of the diaphragmatic defect further supported previous trauma as the mechanism of injury. Our patient’s presentation is particularly interesting considering the lack of TDH reported in the pediatric population, as well as the presenting complaints being primarily gastrointestinal rather than respiratory.

## Introduction

Vague gastrointestinal symptoms such as nausea and abdominal pain are common presenting complaints in the pediatric population, and the list of differential diagnoses is quite long. One specific pathology, the diaphragmatic hernia is most commonly seen as a congenital condition in the pediatric population; however, acquired diaphragmatic hernia is also possible. We discuss a case of a 15-year-old female who presented with such symptoms and who was eventually diagnosed with a left-sided, diaphragmatic hernia resulting from blunt trauma during a motor vehicle accident one year prior to manifesting symptoms. This article was previously presented as a poster at The Tennessee State Pediatric Conference on August 3, 2021 and at the 36th Annual Pediatric Conference at Quillen College of Medicine on August 7, 2021.

## Case presentation

A 15-year-old female presented to the emergency department for persistent nausea and vomiting. She visited an urgent care clinic nine days earlier for the same symptoms and was sent home with Promethazine after a negative urinalysis and rapid strep test. Soon after this, her vomiting resolved. However, the day before presentation to our emergency department (ED), the patient experienced increasing epigastric pain, nausea, and vomiting and went to an outside emergency department. Her work-up at that time included a complete blood count (CBC), comprehensive metabolic panel (CMP), serum lipase, urine pregnancy test, and urinalysis. The results from these studies were all within normal limits. She was given lidocaine and aluminum-magnesium hydroxide, which resulted in the resolution of symptoms, and was discharged with Ondansetron, Omeprazole, and Sucralfate.

In the ED, the patient complained of sharp, persistent epigastric pain, several episodes of coffee ground emesis, decreased ability to tolerate oral intake and decreased urine output. She also reported having fatigue and shortness of breath. When questioned, the patient denied fever, cough, congestion, and rash. She reported no alcohol, drug, or tobacco use. Her only medication use was NSAIDs once every couple of months for headaches. Relevant family history included a maternal history of gastroesophageal reflux disease and a cousin with Crohn’s Disease.

Physical exam showed a tired-appearing, well-developed teenage female. Her vital signs at the time of presentation were a blood pressure of 131/81 mmHg, temperature of 98.9°F, heart rate of 101/min, respiratory rate of 20/min, and oxygen saturation of 97%. The patient had epigastric abdominal tenderness to palpation, but negative costovertebral angle (CVA) tenderness and negative Murphy’s sign. The cardiovascular exam showed mild tachycardia with a regular rhythm. Her respiratory exam showed normal effort and the lungs were clear to auscultation bilaterally.

**Figure 1:**
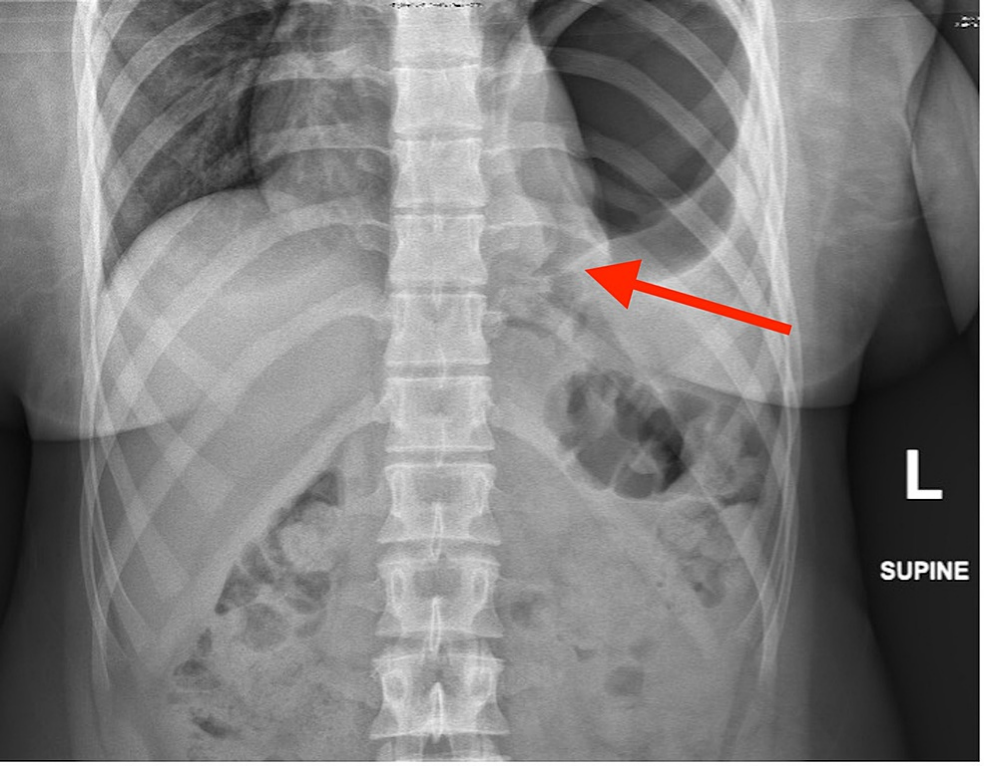
X-ray showing bowel passing through the diaphragmatic defect into thoracic cavity

**Figure 2:**
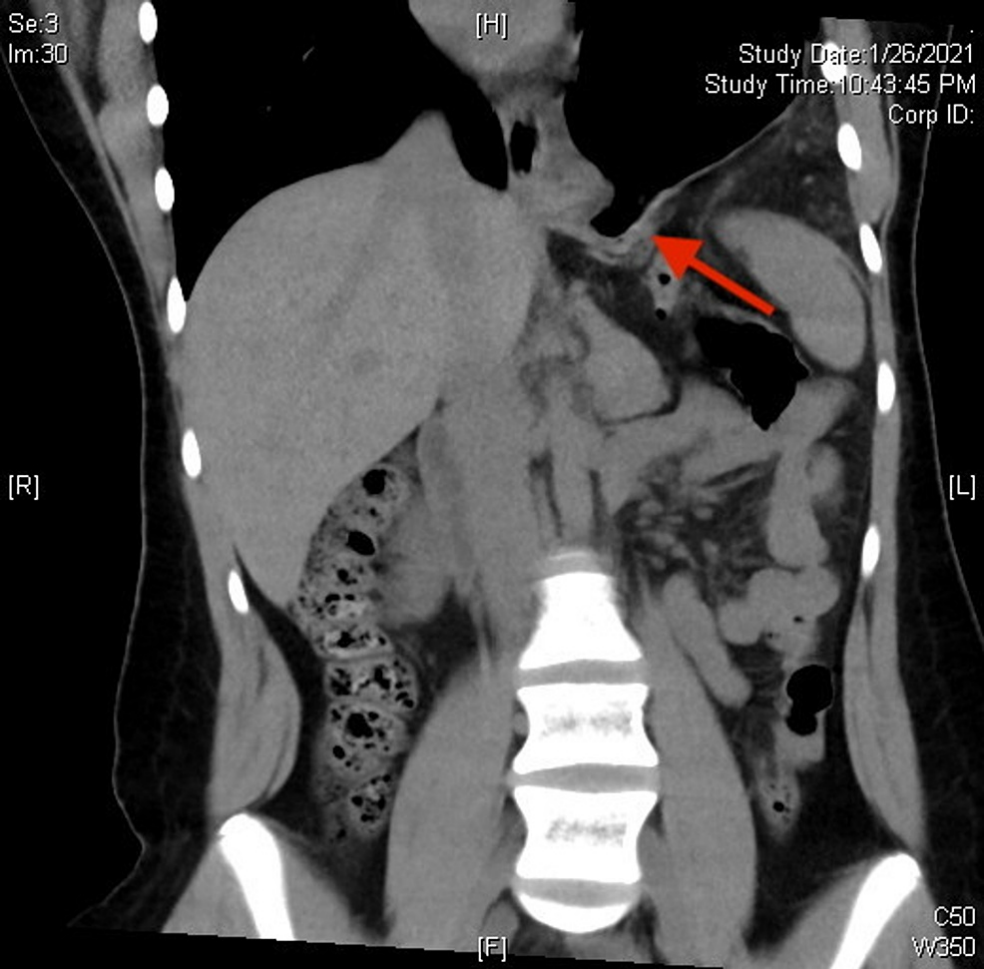
CT scan showing bowel passing through the diaphragmatic defect into thoracic cavity

Initial lab investigation showed no evidence of anemia, a WBC within normal limits, normal serum amylase and lipase, and an unremarkable CMP. An abdominal x-ray showed a large air-fluid level underneath an elevated left hemidiaphragm with left basilar atelectasis (Figure 1). CT scan without contrast was then performed, which revealed a left-sided diaphragmatic hernia with upward herniation of the entire stomach (Figure 2).

Upon further investigation of the patient’s medical history, it was found that the patient presented to the ED about a year earlier for a motor vehicle accident involving blunt injuries to her left chest, left ankle, and head. Physical examination at that time was significant for left-sided chest wall pain predominately along the left lower costal margin. The patient was evaluated with a chest x-ray that showed clear pleural spaces and lungs. There was no diaphragmatic hernia noted on that film. Chart review revealed an upper gastrointestinal series she received as a toddler that showed the stomach in a normal position.

The patient was admitted and started on an intravenous infusion of pantoprazole 40 mg twice daily. Pediatric Gastroenterology and Pediatric Surgery were consulted. The patient underwent a laparoscopic repair of the diaphragmatic defect. During the surgery, the stomach was visualized in the left chest cavity protruding through a large defect measuring approximately 8-10 cm. The defect was found not to involve the gastroesophageal junction; it was found to cross the triangular ligament of the left lateral segment of the liver involving the central tendinous portion of the diaphragm. These findings were most consistent with previous trauma being the mechanism that led to the resulting defect.

## Discussion

We presented a case of traumatic diaphragmatic hernia (TDH) in a 15-year-old female who reported symptoms about a year after the instigating trauma. TDH was first discussed in the literature in 1957 as the increasing incidence of motor vehicle accidents caused an associated increase in the incidence of TDH [1]. It is said to occur in approximately 3% of abdominal injuries [2]. TDH has previously been classified by the mechanism of action: penetrating trauma vs. blunt trauma. Multiple studies have found that the majority of TDH are caused by blunt trauma, with motor vehicle accidents being the most common etiology [2,3]. 

The right hemidiaphragm tends to be less commonly affected due to the protective nature of the liver position [2]. A chest radiograph is the first choice study when evaluating TDH, with asymmetry of the hemidiaphragm being the concerning finding [4]. CT scan is another commonly ordered exam for trauma patients due to its quick availability of results, and its usefulness has progressed due to the increased sensitivity and specificity for detection of TDH with the advent of helical CT scans [5]. The use of MRI was evaluated by Barbiera et al., who noted advantages such as allowing one to better assess the entirety of the diaphragm as well as to more accurately gauge the size of the tear [6]. Although this can be beneficial, the ability to perform an MRI in emergency situations or multi-trauma patients is limited. Shackleton et al. concluded that an algorithm for imaging possible TDH cannot be developed because of the high false-negative rates, in part due to their finding that all their TDH cases caused by penetrating injuries were diagnosed intraoperatively after having nonspecific imaging findings [4].

Research on pediatric TDH is much more limited as it seems TDH is less prevalent in the pediatric population. Marzona et al. found that respiratory concerns are the most common presenting issue in both pediatric and adult patients. Additionally, gastrointestinal symptoms, if present, are more commonly found in adults [7]. This is particularly interesting as our patient was a 15-year-old female who presented with symptoms more likely found in an adult with TDH [7]. While most TDHs present acutely after the inciting trauma, TDH can occasionally present much later, as was the case with our patient. These so-called delayed or chronic TDHs are not common and are usually caused by diaphragmatic tears which are smaller than the tears typically seen in acute TDHs [3,8].

## Conclusions

Our patient is particularly interesting since pediatric TDHs are less common, delayed presentation of TDH is less common, and her presenting complaints were mainly gastrointestinal rather than the more common respiratory complaints. Based on our patient presentation, severe persistent abdominal pain in a pediatric patient paired with a history of major trauma suggests TDH should be considered in the differential diagnosis when there is diagnostic uncertainty. If TDH is suspected, we suggest evaluation using a chest radiograph and confirmation with a CT scan if needed.
